# The ESR Audit Tool (Esperanto): genesis, contents and pilot

**DOI:** 10.1007/s13244-018-0651-0

**Published:** 2018-09-05

**Authors:** European Society of Radiology (ESR)

**Affiliations:** European Society of Radiology (ESR), Am Gestade 1, 1010 Vienna, Austria

**Keywords:** Medical audit D008485, Clinical governance D054976, Clinical audit D054869, Guideline adherence D019983, Radiology D011871

## Abstract

**Abstract:**

Clinical audit is a powerful tool to improve patient care, experience and outcome. It consists of measuring a clinical outcome or procedure against predefined standards, identifying differences between current practice and the standards, and changing practice where necessary to facilitate meeting the standards, followed by re-audit (the audit cycle). The recently implemented European Council Basic Safety & Standards (BSS) Directive (2013/59/Euratom) emphasises that carrying out clinical audit is compulsory in the EU “in accordance with national requirements”. In 2017, the ESR published a Clinical Audit Tool booklet (*Esperanto*), consisting of an explanation of clinical audit, guidance on how to start a program in a radiology department, and 17 sample audit templates which could guide departments through the process of performing audits. A sample of key audits from these templates was performed by participating EuroSafe Imaging Star departments as a pilot project, with participants providing feedback on the audit process, and their experience of using the templates. Responses showed that the chosen topics were relevant, the audit process and templates were straightforward and easy to use, and that the audit process was time-efficient. Following this successful pilot, the Audit Tool has been made available for all potential users through the ESR website.

**Key Points:**

• *Clinical audit is the process of assessing one’s practice against defined standards, altering practice if necessary to meet standards, and re-assessing following changes to confirm improvement*

• *Clinical audit should be part of practice in all radiology departments*

• *Under European Council Directive 2013/59/Euratom, clinical audit is compulsory in the EU “in accordance with national requirements”*

• *The ESR has developed a Clinical Audit Tool (Esperanto) to explain the process and guide departments through sample audit templates*

• *This Clinical Audit Tool was successfully piloted in 2017, and is now available to all users on the ESR website*

## Introduction

As part of clinical governance, healthcare organisations are accountable for continually assessing and, where possible, improving the quality of their services [1]. Clinical audits are, if correctly and professional conducted, an excellent method of assessing performance and guiding possible improvements. Clinical audit is a powerful tool to improve patient care, experience and outcome. These audits consist of measuring a clinical outcome or procedure against pre-defined standards in order to identify differences between current practice and the given standards. Clinical practice can thus be evaluated. If the standard is not achieved, reasons for this are explored, changes are implemented based on the results and a re-audit is carried out to ensure improvement. This methodology is often described in terms of the *audit cycle*.

## Methodology: the audit cycle

Audit simply means comparing an element of clinical practice against an agreed standard. In radiological practice, this might mean what we do, how we do it, what equipment we use, and how we interact with our patients, our colleagues and our environment. To put it another way, audit asks one question: “Are we safe?” Audit uses specific methodology, in which a given performance is compared with a preselected standard. If the standard is not achieved, reasons for this are explored, change is implemented and a re-audit is carried out to ensure improvement. This process can be termed the audit cycle (or helix). The audit should be achievable, local, practical, inexpensive, non-threatening and easy (ALPINE).

Carrying out clinical audit ‘in accordance with national requirements’ is compulsory within the European Union. Previous Directives mandated this, but there is renewed emphasis as a result of implementation of the updated Basic Safety and Standards Directive. This updated BSS Directive (Council Directive 2013/59/Euratom) [2, 3] has major implications for European practice in several areas, including documented justification processes for radiation exposure and dose optimisation. In addition, it requires that ‘clinical audits are carried out in accordance with national procedures’. Clinical audit is central to modern medical practice, involving reflective validation of existing practices, and identification of potential changes and improvements, in the interests of patient safety and better outcomes.

## The ESR perspective

The ESR cooperates with institutions, including the European Commission and the heads of the European Radiation Protection Competent Authorities (HERCA), to ensure that a clinical audit is applied properly to improve quality of patient care in Europe, and also to understand the regulators’ perspective for its efforts regarding audit [4, 5]. In the context of the implementation of the Basic Safety Standards Directive, the ESR works with stakeholders to increase awareness of clinical audit among radiologists and to provide radiology departments with a toolkit to perform audits effectively.

### Esperanto

In 2017, the ESR published a booklet, *Esperanto*, summarising the fundamental ethos and building blocks necessary to begin practising clinical audit (www.myesr/esperanto.pdf). Esperanto itself is a constructed auxiliary language devised by a Polish ophthalmologist named Ludovic Zamenhof. Zamenhof espoused the view that lack of a common language led to conflict between disparate ethnic groups. He suggested that Esperanto should be culturally neutral and simple to learn. It should be learnt in parallel with one’s national language representing a common currency between cultures. The audit booklet’s authors felt that these noble aspirations reflected exactly the underlying ideals behind this project.

## The ESR Clinical Audit Tool

The European Commission published guidelines for clinical audit in 2009, and these were summarised in a statement from the ESR in 2011 [3], which could be considered the basis for *internal clinical audit*. There will be variation in how the requirement for clinical audit will be implemented across Europe, but internal assessment within units or departments, which should employ standard audit methodology, is recommended as a systematic and continuing activity with a significant annual output of departmental audit data. This should be in conjunction with an *external clinical audit* (or regulatory audit), which may be required by national legislation, whereby an external auditing body or auditors visit departments at fixed intervals. In whatever form the new legal framework is implemented by each country, internal clinical audit will help departments to comply with legislation, to monitor their own practice and to be well prepared for any external audit.

In preparation for the implementation of this Basic Safety Standards Directive, the ESR Audit & Standards Subcommittee has developed the ESR Clinical Audit Tool. This is a set of suggested audits that can be easily performed, with accompanying templates indicating the steps required to complete each audit, and the information that should be collected and analysed in each case. These suggested audits are an excellent basis for commencing the practice of clinical audit in imaging departments, and for developing audit in those departments already active in this area. The ESR Clinical Audit Tool is designed to increase awareness of clinical audit among radiologists, and to help them make it part of their departmental work. In addition, it can help to demonstrate to external bodies that their department offers safe, well-documented care.

## The ESR audit pilot project

Experience of clinical audit across Europe is variable. In response to this, the ESR Audit Subcommittee undertook a pilot project in 2017. Concentrating on the key areas of radiation protection and patient safety, 17 key audit topics were described and suggested versions of completed templates were produced for each. This pilot project was designed, firstly, to increase awareness of clinical audit among radiologists, and to help them make it part of their routine departmental work. In addition, participation can help to demonstrate to external bodies that radiology departments offer safe, well-documented care.

Five-star EuroSafe Imaging departments were invited to participate in the pilot. Each participating department was asked to complete five audits that were considered key by the Audit Subcommittee. To assist departments, the ESR Audit & Standards Subcommittee, under the guidance of Prof. Barry Kelly and Dr. Adrian Brady, in collaboration with EuroSafe Imaging, developed and completed a pilot project in 2017 to test the prepared audit templates within the network of EuroSafe Imaging Stars. This project was led by Dr. E. Jane Adam and supported by the ESR Audit & Standards Subcommittee, EuroSafe Imaging and the ESR Office.

Twenty-four “five-star” EuroSafe Imaging departments were invited to participate in the pilot survey.

These were:

**Table:** 

▪ Medical University of Innsbruck, Department of Radiology	Austria
▪ Antwerp University Hospital, Department of Radiology	Belgium
▪ H.-Hart Hospital, Department of Radiology	Belgium
▪ Kuopio University Hospital, Diagnostic Imaging Centre	Finland
▪ European Georges Pompidou Hospital, Department of Radiology	France
▪ University Medical Centre Mainz	Germany
▪ University Hospital of Heraklion, Department of Medical Physics	Greece
▪ Affidea Hungary - Szeged University Centre	Hungary
▪ Mater Misericordiae University Hospital, Department of Radiology	Ireland
▪ Mater Private Hospital	Ireland
▪ Connolly Hospital Blanchardstown	Ireland
▪ University of Pisa, Department of Diagnostic and Interventional Radiology	Italy
▪ Institute of Radiology Catholic University	Italy
▪ Humanitas Research Hospital, Department of Radiology	Italy
▪ IRCCS Ospedale Pediatrico Bambino Gesù	Italy
▪ Cittadella General Hospital - ULSS 15 “Alta Padovana”, Department of Radiology	Italy
▪ Azienda Ospedaliero-Universitaria Meyer	Italy
▪ Sant’Andrea Hospital	Italy
▪ Centro Hospitalar e Universitário de Coimbra	Portugal
▪ Hospital Universitario y Politécnico La Fe	Spain
▪ Hospital Clinic Barcelona	Spain
▪ Erasmus University Medical Centre, Department of Radiology & Nuclear Medicine	The Netherlands
▪ Bravis Medical Centre	The Netherlands
▪ Great Ormond Street Hospital London, Radiology Department	United Kingdom

## Materials and methods

### List of topics

Seventeen audit topics were identified for consideration. These focused on radiation protection and patient safety and were therefore felt to be of prime importance in the practice of clinical audit. They are as follows:

1. What is the departmental mechanism for informed consent?
2. Does the department record statistics on the number of accidental/unintended exposures that occur annually?
3. What is the departmental policy for informing patients that they have undergone an accidental exposure?
4. What is the mechanism for record keeping and retrospective analysis of adverse incidents?
5. What is the mechanism for referring accidental exposure events to the medical physicist expert (MPE) and informing the competent authority?
6. Does the department have criteria for what constitutes an accidental or unintended exposure?
7. If the justification process is delegated to an individual other than a radiologist, has that person undergone appropriate training?
8. What is the departmental mechanism to confirm the non-pregnancy status of female patients?
9. Is there a written protocol for the justification of who is responsible for the justification process?
10. For radiation exposure related to health screening, is there a policy affirming justification by a competent authority?
11. What percentage of studies are justified in advance of being performed?
12. What mechanism exists for contacting referrers to permit pre-exposure justification discussions to occur if necessary?
13. Is there a written protocol for who may be responsible for justification of fluoroscopic/interventional radiological procedures?
14. Is there a written protocol for who may be responsible for justification of CT studies?
15. What mechanism is used to evaluate patient dose in high-dose procedures?
16. How old is the equipment in your department?
17. What percentage of procedures have established dose reference levels (DRL)?

The principal goal of this pilot project was to test the audit templates and to amend them, if needed, based on the feedback received from the project’s participants. Thereafter, the ESR Audit Pack would be made available to European imaging departments.

In the pilot, imaging departments were asked to carry out five specific key audits (numbers 4, 8, 14, 15 and 16) and as many of the other 12 audits they wish to do over a period of 3 months. These five ‘key’ audits were chosen for piloting because they focus on fundamental audit topics likely to be of immediate regulatory relevance to any imaging department.

### The five essential audits are:

- What is the mechanism for record keeping and retrospective analysis of adverse incidents? (4)
- What is the departmental mechanism to confirm the non-pregnancy status of female patients? (8)
- Is there a written protocol for who may be responsible for justification of CT studies? (14)
- What mechanism is used to evaluate patient dose in high-dose procedures? (15)
- How old is the equipment in your department? (16)

## Blank template format

1. Audit title
2. Standard against which the audit topic is to be compared
3. Source of standard
4. Importance
5. Target / compliance percentage to be achieved
6. Item or variable to be audited
7. Method: retrospective / prospective / other
8. Data or information to be collected
9. Sample details
10. Target achieved
  Yes / no / not applicable.
  If no: actual result………………………
11. Action to be taken if the target is not met
12. Timing for re-audit

### Example of completed template (audit topic 8: “What is the departmental mechanism to confirm the non-pregnancy status of female patients?”)

1. Audit title
  What is the departmental mechanism to confirm the non-pregnancy status of female patients?
2. Standard against which the audit topic is to be compared.
  EU Directive
3. Source of standard
  EU 2013/59
4. Importance
  Compulsory: legal requirement
5. Target/compliance percentage to be achieved
  100%
6. Item or variable to be audited
  Request form / order comms
7. Method: retrospective / prospective / other
  Retrospective
8. Data or information to be collected
  Identification of a place on the request form/order comm for the practitioner or operator to record the patient’s date of (first day of) the last menstrual period.
  Ensure that the data have always been entered.
9. Sample details
  One month review of request forms/order comms
10. Target achieved
  Yes / no / not applicable
  If no: actual result………………………
11. Action to be taken if the target is not met
  Amendment to include place for these data on the request form.
  Appropriate training to ensure that the data are always recorded.
12. Timing for re-audit
  One year

## Results

The project began on 30 May and concluded in October 2017. Seventeen of the 24 centres (71%) completed the five audits.

In brief, the responding departments felt that:

1. The chosen key topics are relevant
2. The audit process is straightforward to conduct
3. The audit templates are straightforward
4. The correct standards are utilised
5. The process is time-efficient

The results are summarised in Table 1.

**Table 1:** Summary of the Esperanto pilot project results

Audit number	Relevance of topic? (1 = not relevant, 10 = essential)	How difficult was the audit to conduct? (1 = easy, 10 = very difficult)	Correct standard applied? (Percentage of respondents who agreed)	How easy was it to use the template? (1 = simple, 10 = impossible)	Time consuming? (1 = not at all, 10 = very time consuming)
4	9	5	88	3.7	5
8	9.3	3.8	100	4.3	4.6
14	8.5	5.2	76	4.1	4.7
15	9.3	4.5	82	3.9	4.8
16	8	4	82	3.7	4.1
Average	8.82	4.5	85.6	3.94	4.64

All of the materials described (the Esperanto booklet, the suggested blank and completed templates and the pilot results) are available on the ESR website (www.myesr/esperanto.pdf).

The clinical audit initiative is considered to be organic, in other words, evolving, with new topics being constantly added. The authors were keen to emphasise that the templates suggested are not prescriptive, simply one way to approach the subject. Individuals completing audits may find that different methodologies and templates better suit their needs. The templates available are simply one form of foundation.

## Conclusions

Clinical Audit is a valuable tool to measure a department or individual’s activity compared to a pre-defined standard. The most current BSS Directive (Euratom 2013/59) emphasises the mandatory nature of this activity. Our pilot study with its simple Esperanto booklet and attendant templates has shown itself to be robust and simple to use. ESR has made these tools available to all imaging departments (https://www.myesr.org/media/2835) and we hope, as the resources evolve, that they will be a valuable resource for radiology departments, especially with regard to radiation protection and patient safety.

We hope that departments using the ESR Audit Tool will find that it encourages a culture of continuing clinical audit and self-improvement, and that it will provide training to allow departments design and conduct audit on other topics in the future, according to their own local needs and interests.
